# Case study of cervical cancer prevention in two sub-Saharan African countries: Rwanda and Sierra Leone

**DOI:** 10.3389/fmed.2022.928685

**Published:** 2022-09-15

**Authors:** Mohamed S. Bangura, Yuqian Zhao, Maria Jose Gonzalez Mendez, Yixuan Wang, Salah Didier Sama, Kunpeng Xu, Ran Ren, Li Ma, You-Lin Qiao

**Affiliations:** ^1^School of Public Health, Dalian Medical University, Dalian, China; ^2^Sichuan Cancer Hospital and Institute, Sichuan Cancer Center, School of Medicine, University of Electronic Science and Technology of China, Chengdu, China; ^3^Department of Cardiology, First Affiliated Hospital of Dalian Medical University, Dalian, China; ^4^Department of Quality Management, Dalian 3rd People's Hospital, Dalian, China; ^5^School of Population Medicine and Public Health, Peking Union Medical College, Beijing, China

**Keywords:** cervical cancer, vaccination, screening, Rwanda, Sierra Leone

## Abstract

**Background:**

Cervical cancer is a public health issue of global concern. It is a preventable disease but continues to threaten the lives of women, especially in developing countries in sub-Saharan Africa.

**Methods:**

We selected two African countries in sub-Saharan Africa (the Republic of Rwanda and the Republic of Sierra Leone) to show a good example of cervical cancer prevention and constrains hindering countries from effectively implementing cervical cancer programs. Secondary data were collected from the World Health Organization (WHO), the International Agency for Research on Cancer (IARC), the Global Burden of Cancer (GLOBOCAN), the United Nations Development Programme (UNDP), and the World Bank and from official websites of the selected countries. A descriptive analysis method was used to source data and compare variables such as the associated factors, disease burden, prevention programs, health workforce, success factors, and challenges.

**Results:**

Rwanda achieved 93.3% human papillomavirus (HPV) vaccination of the three doses vaccinating girls in class 6, as a result of effective school-based platform delivery system and community partnership to identify girls who are out of school. Rwanda reduced the historical two-decade gap in vaccine introduction between high- and low-income countries. The country also introduced a nationwide cervical cancer screening and treatment program. An impressive decreased cervical cancer incidence rate in Rwanda in recent years was observed. Sierra Leone lags behind in terms of almost all cervical cancer prevention programs. Therefore, Sierra Leone needs more efforts to implement cervical cancer intervention programs at the national level, including HPV vaccination, and train and increase the number of health professionals, treatment, and palliative care services to accelerate cervical cancer activities.

**Conclusion:**

The disease burden of cervical cancer for Rwanda and Sierra Leone is heavy. There remains huge room for improvement in preventing and controlling cervical cancer in these countries. The goal of cervical cancer elimination would not be feasible in countries without the awareness and will of the policymakers and the public, the compliance to fund cervical cancer programs, the prioritization of cervical cancer activities, the availability of resources, the adequate health workforce and infrastructure, the cross-sectional collaboration and planning, inter-sectorial, national, regional, and international partnerships.

## Introduction

Cervical cancer is a public health issue of global concern, with a projected number of 604,127 new cases and 341,831 deaths worldwide in the year 2020 (1). It is a preventable disease, but it continues to threaten the lives of women in their prime stage of life, especially in developing countries such as those in sub-Saharan Africa (2, 3). A recent study showed that cervical cancer accounts for 13% of female cancers. The study also illustrated Eastern and Western Africa as high-risk regions, with a cumulative risk of 3.8%, as well as (2.9%) Southern Africa (4). Many high-income countries decreased cervical cancer cases by more than 70% in the late 1950's to 1960's, by implementing cervical cancer screening programs (5). However, the situation is the opposite in developing countries, mainly in sub-Saharan Africa, due to the lack of screening, treatments of pre-cancerous lesions, limited resources, and other barriers; many sub-Saharan African countries have been unable to achieve cervical cancer rate reduction compared with many developed countries (6–10).

The World Health Organization (WHO) proposed an intermediate target toward elimination of cervical cancer by 2030, namely, 90% of girls fully vaccinated with the human papillomavirus (HPV) vaccine by the age of 15 years, 70% of women screened using a high-performance test by the age of 35 years and again by the age of 45 years, and 90% of women with pre-cancer treated and 90% of women with invasive cancer managed. However, women in many sub-Saharan African countries face obstacles such as lack of funding and access to healthcare facilities due to geographic constrains. The challenges to introduce cervical cancer screening and HPV vaccination in sub-Saharan Africa are related to the neglect to implement the prevention program, shortage of personnel, cost, weak infrastructure system, insufficient technical staff, and lack of prioritization and will to support cervical cancer programs (11).

Furthermore, many sub-Saharan African countries are constrained by limited resources, some of them are yet to get access to the HPV vaccine, and some are yet to start any HPV demonstration project at national and/or district pilot phase. Although some countries have vaccination programs, they are below the recommended vaccination coverage.

The aim of this analysis was to describe cervical cancer disease burden and trends, HPV vaccination, screening, and health-related resources in Rwanda and Sierra Leone, to provide information and suggestion for sub-Saharan Africa countries on elimination of cervical cancer.

## Methods

### Country selection

**Figure 1:**
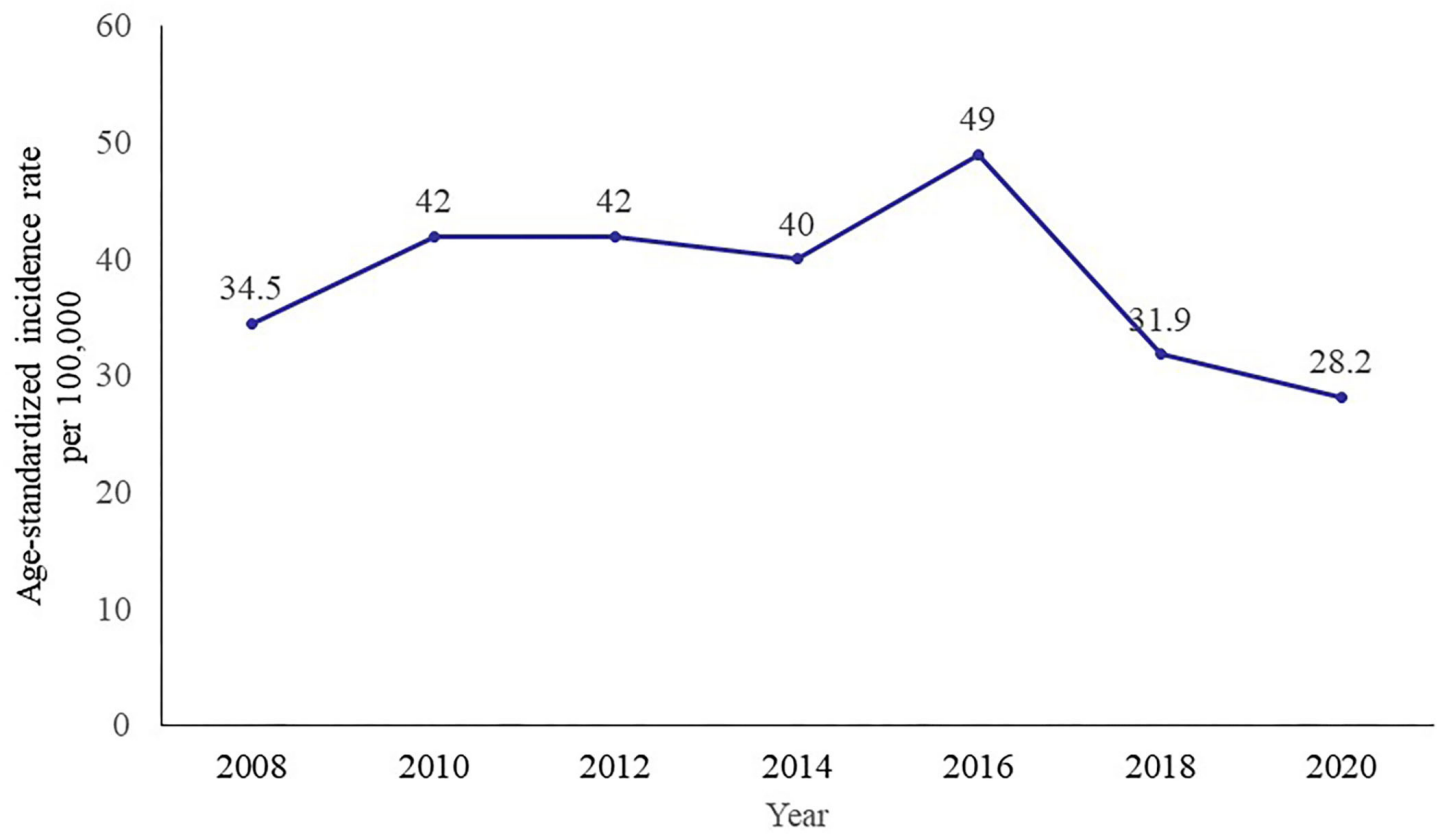
Trend of age-standardized incidence rate of cervical cancer in Rwanda, 2008–2020. Data source: IARC.

We selected two African countries in sub-Saharan Africa, the Republic of Rwanda (Rwanda) and the Republic of Sierra Leone (Sierra Leone), to describe the possibilities and challenges of carrying out cervical cancer prevention programs. We chose Rwanda for study because of its thriving cervical cancer prevention programs, such as the mass nationwide HPV vaccination program, the number of referral hospitals for cervical cancer management, the encouraging number of oncologists, physicians and nurses, palliative care services, vaccine platform delivery systems (community-based and private partnership), and cervical cancer mobile team outreach programs in remote areas. A decreased cervical cancer incidence rate in Rwanda in recent years was observed recently (Figure 1). In terms of communicable diseases, Rwanda has reduced the burden and has a strong public health system compared with other sub-Saharan African countries. On the contrary, Sierra Leone had only done pilot phase of cervical cancer demonstration at the district level since 2014 and has and/or among countries with the worse resources constrains in the subregion in terms of cervical cancer of cervical cancer program implementation. In addition, the first author, Bangura Mohamed S, who is from Sierra Leone, aims to make a difference in cervical cancer prevention and control in his country. In short, Rwanda and Sierra Leone represented the best and worst cervical cancer prevention program implementation among sub-Saharan Africa, respectively. Other sub-Saharan African countries mostly fall between these two countries.

### Data collection

Secondary data were collected from the WHO, the International Agency for Research on Cancer (IARC), the Global Burden of Cancer (GLOBOCAN), the United Nations Development Programme (UNDP), and the World Bank, and official websites of the governments of the analyzed countries. The search and data collection was performed by two researchers (MSB and MJGM) independently and then were presented and discussed with the study team.

We used a descriptive analysis method to source data, describe the results, and list the variables such as the prevalence of known associated factors of cervical cancer, cervical cancer incidence and mortality rates, HPV vaccination coverage, cervical cancer screening, implementation programs, challenges and other indicators. The validity or trustworthiness of the organizations that managed and released the data enabled our team to reliably use the data for this study.

### Data analysis

The indicators were divided into two category: general indicators and cervical cancer-specific indicators. General indicators included gross domestic product (GDP), health systems, the maternal mortality rate (MMR), health workforce, health expenditure, and infrastructure, which were compared with the international standard recommended number of physicians per 10,000 population, the number of gynecologists and oncologist nurses, and expenditure in healthcare services. The cervical cancer indicators included the prevalence of associated factors and cervical cancer incidence and mortality rates, cervical cancer nationwide screening activities implemented, and HPV vaccination coverage in these countries, which were compared with the WHO- and GAVI-recommended thresholds.

We used the descriptive method and direct comparative method to assess what are lacking and/or present and absent in the countries in terms of cervical cancer programs. We used direct observation and also compared the outcomes of these countries with international standard thresholds.

No human studies, animal studies, or potentially identifiable human images or data are presented. The study was approved by the Chinese Academy of Medical Sciences & Peking Union Medical College (No. DL2021194001L).

## Results

### General description of the included countries

#### Rwanda

Rwanda is a landlocked country in sub-Saharan Africa (in Eastern Africa) with a population of 11 million comprising 2.72 million women, at the age of 15 years and older, who are at risk for developing cervical cancer (12). The country has robust record of tackling and curbing communicable and non-communicable diseases. The MMR of Rwanda has been reduced to an accelerating number from 1,071 per 100,000 women in 1992 to 203 in 2020, drawing closer to the Sustainable Development Goals commitment number of 70 by 2030 (13). The key indicators of the country are as follows: health financing, with the total health expenditure per capita was $58.31 in 2018 and out-of-pocket total expenditure on health in 2011 was 21%; the GDP per capita in 2011 and 2012 were $668.5 and $704.2, respectively (Table 1); the health workforce such as physicians per 100,000 population was 0.06 in 2010; the ratio of doctor to population was 1:16,001; the ratio of nurses and midwives per 100,000 per population was 1:1,291 in 2010; the antenatal care coverage population with at least four visits was 43.90% in 2021; the population with household spending on health >10% of the total household budget (SDG 3.8.2) was 1.15% in 2021; and the Human Development Index (HDI) was 0.43 (14).

**Table 1:** General indicators of Rwanda and Sierra Leone.

**Indicators**	**Country**	
	**Rwanda**	**Sierra Leone**
GDP per capita ($)/year	797.9/2020	509.4/2020
Health expenditure (% of GDP)/year	6.41/2019	8.75/2019
MMR^*^/year	248/2017	1,120/2017
Doctors per 10,000/year	1.18/2019	0.74/2018
Number of oncologists (*N*)	5	1
Number of oncologist nurse (*N*)	19	ND
Number of palliative staff (*N*)	75,000	–
Number of referral hospitals (*N*)	100	–
Cervical cancer screening scale/year	Nationwide/ 2011-2012	Pilot stage /2014

* MMR, maternal mortality rate, per 100,000; ND, no data; -, unavailable.

Data source: the World Bank, Rwanda Biomedical Center, and UNDP.

Rwanda met the Global Alliances for Vaccines and Immunization (GAVI) criteria, with a DTP3 threshold of 70% national coverage, and a pilot demonstration was carried out to check the ability to deliver complete multi-dose series of vaccines to at least 50% of the target population in a district within the country to determine countries qualification for vaccination assistance (15). As a result of robust national health system of Rwanda, over 90% of all Rwandans infants within the age of 12–23 months received all the WHO-recommended basic immunization.

#### Sierra Leone

Sierra Leone is located in Western Africa. According to the World Bank, the life expectancy in Sierra Leone has increased from 39 to 54 years from 1990 to 2017, being the fourth lowest worldwide. The general government expenditure on health as a percentage of total of government was 10.84% in 2014. The per capita GDP of Sierra Leone in 2020 was 509.4$. The ratio of medical doctors per 10,000 people was 0.74. According to the Demographic Health Survey (DHS), the number of nurses and midwives personnel per 10,000 was 72.54 in 2018. The percentage of births by 15- to 19-year-olds attended by skilled health personnel in 2017 was 81.72% (78.28–84.72). According to the latest information, the antenatal care coverage (at least four visits) is 78.8% (16). Sierra Leone has the highest MMR in the world, at 717 maternal deaths per 100,000 live births in 2019 (17). Sierra Leone continues to experience outbreaks that have overwhelmed and added to the already fragile health system (16). In addition, the per capita total expenditure on health services is 95$, and 76% of health spending is from individual out-of-pocket expenditure, 16% is government-funded, and 13% is from donors' fund (18).

### Rwanda indicators

#### Cervical cancer disease burden in Rwanda

Cervical cancer is common and fatal among women in Rwanda. Eastern Africa is one of the most affected cervical cancer burden region, with an incidence of 30 cases per 100,000 women per year (19). Before 10 years, the global cancer statistics showed that 1,000 women are diagnosed annually in Rwanda, and almost 700 women died of cervical cancer in the year of 2010 (4). The disease is the most commonly diagnosed condition among women aged 15–44 years (20). The estimated incidence of cervical cancer in Rwanda was 49 cases per 100,000 women per year, which is higher than the estimated cases for Eastern Africa and globally, with 34.5 and 16 new cases around 2010, respectively. Studies showed that the prevalence of pre-cancer and invasive cervical cancer (5.9%) and (1.7%) was high in Rwanda. The associated factors with the high prevalence were a result of the factors such as multiple sexual partners, high parity (over three kids born), tobacco use, and long-term contraceptive pill use (21, 22). Table 2 shows the data of the associated factors of cervical cancer by country.

**Table 2:** Cervical cancer-associated factors among women in Rwanda and Sierra Leone.

**Factors**	**Rwanda**	**Sierra Leone**
**Smoking**		
Smoking of any tobacco adjusted prevalence (%) [95% UI]^*^	4.2 [2.5–6.3]	8.6 [5–13]
**Parity**		
Total facility rate per woman (*N*)	3.8	4.3
**Hormonal contraception (%)**	8.40	-
Oral contraceptive use among married women or in union (%)	-	5.30
Injectable contraception use among married women or in union (%)	-	11.9
Implant contraceptive use among women who are married or in union (%)	-	3.60
**HIV**		
Estimated percent of adults aged 15–49 living with HIV (%) [95% UI]	3.2 [2.6–3.6]	1.8 [1.5–2.3]
Estimated percent of young adults aged 15–24 living with HIV (%) [95% UI]	-	1.1 [0.5–1.8]
HIV prevalence among sex workers (%)	-	6.69
Estimated number of adults (15+ yrs) living with HIV (*N*) [95% UI]	-	38,000 [31,000–47,000]

-, no data available/not applicable.

* Smoking at the time of the survey, including both daily and occasional smoking.

Data source: the World Bank and WHO.

Recent data showed that the current (2020) cervical cancer burden in Rwanda is still high (23). Approximately 1,229 new cervical cancer cases are diagnosed yearly, and the data showed that the crude incidence rate is higher in Rwanda (18.7 per 100,000) than the world crude incidence rate (15.6 per 100,000). However, the age-standardized incidence rate is half the Eastern Africa age-standardized rate (28.2 vs. 40.1 per 100,000), although still higher than the world age-standardized rate (13.3 per 100,000). The cumulative risk (3.04%) for women in Rwanda at 75 years old is higher than the world cumulative risk (1.39%; Table 3) (24). Cervical cancer ranked the second most common cancer in Rwanda. In 2020, 829 cervical cancer deaths occurred, and it ranked the first leading cause of female cancer death in Rwanda. The crude mortality rate, age-standardized mortality rate, and cumulative risk at 75 years old are all higher than the world rates. While there are little differences in terms of rates between Rwanda and Eastern Africa (Table 3) (25).

**Table 3:** Cervical cancer incidence and mortality in Rwanda, Sierra Leone, sub-Saharan Africa, and worldwide estimates for 2020.

**Indicator**	**Rwanda**	**Eastern Africa**	**Sierra Leone**	**Western Africa**	**World**
**Incidence**					
Annual number of new cancer cases (N)	1,229	54,560	504	27,806	604,127
Crude incidence rate^*^	18.7	24.3	12.6	13.9	15.6
Age-standardized incidence rate^*^	28.2	40.1	21.2	22.9	13.3
Cumulative risk at 75 years old^**^ (%)	3.04	4.46	2.48	2.48	1.39
**Mortality**					
Annual number of new cancer deaths (N)	829	36,497	367	18,776	341,831
Crude mortality rate^*^	12.6	16.3	9.18	9.41	8.84
Age-standardized mortality rate^*^	20.1	28.6	16.4	16.6	7.25
Cumulative risk (%)at 75 years old^**^	2.27	3.36	1.99	1.88	0.82

* Rate per 100,000 women per year.

** Cumulative risk (incidence/mortality) is the probability or risk of individuals getting the disease in ages 0–74 years. For cancer, it is expressed as the % of newborn children who would be expected to develop or die of a particular cancer before the age of 75 years if they had the rates of cancer observed in the absence of competing causes.

Data source: the IARC.

#### Human resource training and infrastructure

As part of the human resource training to recruit competent personnel in the healthcare sector, the Rwanda National University of Medicine started a 4-year residency program in pathology; 12 pathologists have graduated and more had been trained in 2018. In 2013, community health workers engaged in doing mobile team work with one physician and four nurses engaged in visiting local health centers for free 3-day HPV screening (26). Rwanda initiated a policy to introduce 45,000 community health workers to provide essential services. In addition, the Ministry of Health of Rwanda has trained 30,000 new community health workers in palliative care at the community level for HIV and cancer and other chronic disorder management (27).

A comprehensive cancer center was set up at the district hospital level in 2012. A majority of the referral hospitals have maintained cancer registries. By 2013 to 2015, cervical cancer screening in Rwanda was decentralized into 30 public hospitals and nearly 100 health centers by 2015 (26).

#### Achievement of Rwanda in cervical cancer prevention

Rwanda was the first country in Africa to develop, introduce, and implement a nationwide strategies for cervical cancer prevention, control, and treatment. The country demonstrated effective cervical cancer program planning and introduction and serve as an ideal example of human resources for health personnel and healthcare settings in Africa or in developing regions.

Rwanda was recorded as the first developing country in the world to offer free universal HPV vaccination (28). A nationwide sensitization campaign preceded the delivery of the first dose. In 2011, the Ministry of Health of Rwanda partnered with international donors to offer HPV vaccines for free to girls under the required ages. This vaccination introduction was successful as a result of the Ministry of Health partnering with public–private and community collaboration to promote effective, efficient, and equal vaccine and vaccination delivery nationwide. This was as a result of effective school-based platform delivery system and community partnership to identify girls who are out of school (28). From 2011 to 2012, Rwanda recorded 227,246 vaccinated girls with three doses of the HPV vaccine with the three dose coverage rates of 93.2 and 96.6% achieved in 2011 and 2012, respectively (Table 4).

**Table 4:** HPV vaccination coverage in Rwanda, 2011–2012.

	**2011**			**2012**		
	**Round 1**	**Round 2**	**Round 3**	**Round 1**	**Round 2**	**Round 3**
**No. of girls vaccinated**						
In school^*^	91,752	89,704	88,927	137,147	13,645	134,115
Outside school	2,136	3,066	3,180	1,162	845	1,024
Overall	93,888	92,770	92,107	138,309	135,490	135,139
Cumulative coverage (%)^**^	95.0	93.9	93.2	98.8	96.8	96.6

* Three rounds of vaccination in 2011 only covered girls who were in grade 6 of primary school, whereas the rounds in 2012 covered girls who were then in grade 6 of primary school or the 3rd year in secondary school.

** The denominator for 2011; 98,792 eligible girls; denominator for 2012:139,968 eligible girls.

The country also introduced nationwide cervical cancer screening and treatment programs based on visual inspection with acetic acid (VIA), testing for HPV DNA, cryotherapy, loop electrosurgical excision procedure, and other advanced treatment options (26). As of 2021, Rwanda has screened 16,563 and 559 women treated for pre-cancerous lesions.

Rwanda reduced the historical two-decade gap in vaccine introduction between high- and low-income countries. High coverage rates were achieved due to a delivery strategy that built on Rwanda's robust vaccination system and human resource framework. Those great efforts and investments in healthcare and cervical cancer prevention led to great achievement. An impressive decrease in the cervical cancer incidence rate in Rwanda in recent years was observed (Figure 1) (24).

### Sierra Leone indicators

#### Cervical cancer disease burden in Sierra Leone

Sierra Leone has a female population of 2.23 million between the ages of 15 years and above who are at risk of developing cervical cancer. Data are yet to be available on HPV burden in the general population. However, in Western Africa region where Sierra Leone is located, an estimate of 4.3% of the women in the general population of the country harbor cervical HPV16 and18 infections, and 55.6% of invasive cervical cancers are attributed to HPV 16 and 18, respectively (29). An estimate of 504 new cervical cancer cases are diagnosed, and 367 cervical cancer deaths occur annually in Sierra Leone. According to estimation for 2020, the age-standardized incidence is estimated above 25.6 per 100,000 women. Cervical cancer ranks the second leading cause of female cancer occurrence and cancer death, and it is the second commonest female cancer and leading cause of cancer death among women aged 15 to 44 years. Table 3 shows the new cases and deaths of cervical cancer in Sierra Leone, Western Africa, and the world. According to recent data, there is no cancer registry available in Sierra Leone yet. The crude incidence rate, age-standardized rate, and cumulative rate among 75-year-old women are almost equal to those of Western Africa and, in some cases, higher than the world rates. This may be attributed or linked to the absence of cervical cancer screening and HPV vaccination programs (30).

Studies have shown some of the exact variables, such as sexual behavior, contraception use, parity, HIV, and smoking, as key behavior risk factors of cervical cancer among women (31–34). The study showed that the rate at which women smoke in the country is high, and parity and estimated HIV prevalence among women are also high.

#### Human resource training and infrastructure

Sierra Leone has a shortfall, unequal and maldistribution of its health workforce. An estimate of 92% of the doctors and 72% of the nurses reside in the urban settings, where only 18% of the population of the country is located. According to recent findings from the Global Disease Burden study, statistics showed that Sierra Leone is one of the countries with the least workforce in sub-Saharan Africa and probably in the world. Sierra Leone has the highest workforce attrition, dropout, and health personnel switching rates compared with other public health sectors. If the country is to tackle this situation, it estimated to spend about $18.25 per capital to reach similar workforce retention in Guinea and Liberia, neighboring countries (35–37). In 2017, Sierra Leone had only 300 midwives to serve a population of seven million people (36); however, an increase of 987 midwives was recorded in 2019 (17). The health indicators such as the total number of oncologists, number of oncologist nurses, number of trained health staff to provide palliative services, and number of cervical cancer referral hospitals to manage the disease are presented in Table 1 (26, 27, 38, 39).

#### Cervical cancer prevention in Sierra Leone

The country has implemented the One Health approach at national and regional levels to enhance coordination of multi-sectorial response to health, but the One Health approach and/or national action plan did not capture specific policy for scaling up trainings, personnel, and education programs for cervical cancer activities (18). In addition, in 2021, the UNFPA-Sierra Leone and the University of Sierra Leone Teaching Hospitals Complex (USLTHC) established the Public–Private Partnership Pilot Program on Cervical Cancer Screening and developed 3 first ever national policy on cervical cancer, strategic plan, and clinical guidelines for the management of cervical cancer and refurbished reproductive health centers in seven health centers. In 2020, China and the UNFPA-Sierra Leone partnered on reducing maternal mortality support training of 50 healthcare providers on cervical cancer screening and management of cervical cancer pre-cancerous lesions (17), but the number is still low compared to the population (2.23 million) of women in Sierra Leone who are at high risk of HPV infection (29).

Sierra Leone is one of the countries eligible for GAVI funding to introduce HPV vaccination and cervical screening interventions in Africa. The burden of cervical cancer is high, although in August 2021, there was yet another national HPV vaccination program. The country has completed national demonstration projects in 2013–2014, supported by GAVI. Sierra Leone projected to add HPV vaccine into the routine immunization schedule after a national pilot phase in 2021, and GAVI projected to support HPV vaccine introduction in Sierra Leone by 2023 (40).

Some low-income countries and regions have used VIA as a method and other low-cost strategies in reducing the rate of cervical cancer (41). However, the situation in Sierra Leone is different. Previous findings showed that cervical cancer screening is unavailable in Sierra Leone, except demonstration projects conducted in a region using VIA in 2012–2014 (30). In general, Sierra Leone is lagging behind in terms of cervical cancer screening program.

## Discussion

The primary purpose of comparing indicators from both countries was to analyze the significant and likelihood of reducing cervical cancer in these sub-Saharan African countries. Rwanda has performed remarkably well in implementing HPV vaccination and nationwide cervical cancer screening activities, and has increased the number of referral hospital health workforce to manage cervical cancer patients, which was as a result of developmental improvements such as health financing system, health policy, an increase in investments in the infrastructural system with good referral systems from remote areas to urban centers, mass training of medical staff, and high turnout of medical graduates (26). In sub-Saharan Africa, Sierra Leone is one of the countries with the lowest (0.2%) health workforce and shortage of healthcare personnel and encounters constraints of sanitation facilities and access to healthcare. Sierra Leone is lagging behind in terms of almost all cervical cancer prevention programs. In order to achieve health and development, reports showed that improving the GDP of a country is critical (42–47). Both Rwanda and Sierra Leone have huge potential to attain an incidence rate of <4 cases per 100,000 women per year, if cervical cancer programs in these countries, especially in Sierra Leone, are given attention to.

### Strategies for preventing and controlling cervical cancer in Rwanda and Sierra Leone

The current cervical cancer situation in sub-Saharan Africa poses a challenging health outcome for these countries. To reduce cervical cancer disease burden, it would be necessary to implement the three WHO multiple spring pillar strategies, namely, 90% of girls fully vaccinated by age 15 years, 70% of women screened with a high-performance test by age 35 and 45 years, and 90% of women with pre-cancer treated and those with invasive cancer managed.

In countries with high coverage of HPV vaccination as in Rwanda, they should maintain the ongoing efforts to monitor cervical cancer incidence and mortality, assess the impact of HPV vaccination programs, and also optimize high service quality. In recent years, the WHO recommended a two-dose HPV vaccination schedule (48). In the year of 2022, the WHO Strategic Advisory Group of Experts on Immunization (SAGE) evaluated evidence and recommends updating dose schedules for HPV that one- or two-dose schedules for the primary target of girls aged 9–14 years (49). The recommendation of the single-dose strategy could accelerate progress toward the goal of vaccinating 90% of girls younger than 15 years by 2030. Rwanda should closely follow the recommendations from the WHO, saving its limited health resources, and, at the same time, take the advantage of its robust vaccination system to maintain sustainability of the high coverage of vaccination programs.

Meanwhile, efforts on the cervical cancer screening program for early diagnosis and treatment are necessary. For example, a successful 5-year cervical cancer screening program in Inner Mongolia, China, screening over 40,000 women demonstrated a clear practical scenario of how to implement screening programs in low-resource settings as a way to reduce cases and promote women health and wellbeing in a developing country context (50). Furthermore, data from China showed that using low-resource screening methods (VIA/VILI and careHPV testing methods) is feasible, acceptable, and efficient in detecting cervical cancer and pre-cancerous lesions (41, 51). These strategies may be suitable for Rwanda to incorporate into its screening programs and healthcare system and to provide a lifetime screening service for adult women, rather than copying the high-resource intensive liquid-based cytology screening program, which is costly and impossible to sustain in resource-limited countries and regions. And if possible to source more in the area of knowledge and technology transfer to sub-Sahara African countries in the area of the screening material such as careHPV or Artificial Intelligence (AI) technologies from other countries; which will increase screening coverage of women and it works well in low resource settings with limited health infrastructure. In addition, limited data for pre-cancer and invasive cancer treatment and management were available; therefore, further investigation on this part should be noticed. Although there has been remarkable progress, substantial activities are still needed to further decrease cervical cancer burden in Rwanda (52).

Sierra Leone needs total commitment from the policymakers and public will to prioritize and fund cervical cancer activities in the country. Sierra Leone mainly needs to give attention to cervical cancer prevention activities. This attention can be in the form of providing sustainable funding and investment in primary healthcare facilities; training more health professionals; increasing the capacity of the health workforce and cancer registry; increasing funding for cervical cancer activities; creating local, regional, and international partnerships; increasing specialist training on oncology; and incorporating cervical cancer interventions into the already exiting health program in the primary healthcare setting. For low-resource settings like Sierra Leone, it is yet to vaccinate girls with the HPV vaccine as did Rwanda with over 93% vaccination coverage. The prioritization of HPV vaccination programs is needed to foster cervical cancer interventions in Sierra Leone as the first step. The recommendation of one-dose schedule is an opportunity for countries like Sierra Leone to meet the stage goal of vaccinating 90% girls for cervical cancer elimination. For cervical cancer screening in Sierra Leone, low-cost primary screening approaches, such as careHPV testing and VIA/VILI, may be feasible if the healthcare workforce improves, to provide the minimal screening service for 70% of women at 35 years of age and again by 45 years of age, as recommended by the WHO.

In general, to further accelerate the 90-70-90 target to eliminate cervical cancer, Rwanda needs to maintain the high HPV vaccination coverage for girls and increase screening coverage, and investigation of the cervical cancer treatment and management data for pre-cancer and invasive cancer are needed to assess the treatment rate. Sierra Leone needs to introduce nationwide cervical cancer prevention programs and increase and continue to scale up knowledge and awareness campaigns and HPV vaccination coverage, screening, and treatment.

### Study implications and recommendations

The absence of adequate attention for women's health has posed as barriers for effective cervical cancer prevention programs. The unavailability of screening program such as cytology, HPV testing, VIA/VILI, and LBC due to the technical and financial constraints to organize and fund screenings in the country has posed as a challenge to preventing cervical cancer. Because of the financial and health resource constrains involved to run and maintain sophisticated screening tests such as cytology or to implement organized screening, VIA/VILI, and careHPV testing, other low-resource screening approaches are suggested to be introduced in order to decrease cervical cancer burden.

In addition, it would be necessary to integrate cervical cancer screening and vaccination into existing health programs. For example, Sierra Leone should try to follow Rwanda system in cervical cancer programs such as introducing nationwide HPV vaccination campaign, effective advocacy, and partnering with international and private sector organizations.

Rwanda stands out as a model country for effective cervical cancer prevention and controlling activities in sub-Saharan Africa and other developing countries worldwide. However, Rwanda should still continue increasing the screening coverage, and diagnostic and treatment investments to reach the elimination stage within the twenty-first century.

## Conclusion

The disease burden of cervical cancer in Rwanda and Sierra Leone are heavy. There remains huge room for improvement in preventing and controlling cervical cancer in these countries. The goal of cervical cancer elimination would not be feasible in countries without the awareness and will of the policymakers and the public, the compliance to fund cervical cancer programs, the prioritization of cervical cancer activities, the availability of resources, the adequate health workforce and infrastructure, the cross-sectional collaboration and planning, and inter-sectorial, national, regional, and international partnerships. It is essential to have national well-planned strategies backed by international support in place, then national coverage for HPV vaccination and screening for cervical cancer can be achieved within the shortest possible time in countries such as Sierra Leone and accelerate Rwanda's cervical cancer controlling and preventing progress to meet WHO's agenda and 2030 target to eliminate the disease.

## Data availability statement

The original contributions presented in the study are included in the article/supplementary material, further inquiries can be directed to the corresponding authors.

## Author contributions

MB: conceptualization, literature search, data collection and interpretation, data analysis, manuscript preparation, manuscript editing, and approval of the final draft. MG: conceptualization, literature search, data collection and interpretation, data analysis, manuscript editing, and approval of the final draft. YZ, YW, and SD: literature search, data collection, manuscript preparation, manuscript editing, and approval of the final draft. KX: literature search, data collection, data analysis, manuscript preparation, manuscript editing, and approval of the final draft. LM, RR, and Y-LQ: conceptualization, literature search, supervision, data collection and interpretation, data analysis, manuscript preparation, manuscript editing, and approval of the final draft. All authors contributed to the article and approved the submitted version.

## Funding

This study was supported by the Research, Development and Application of Comprehensive Prevention and Control Technology for Woman Cancer in Countries along the Belt and Road (No. DL2021194001L).

## Conflict of interest

The authors declare that the research was conducted in the absence of any commercial or financial relationships that could be construed as a potential conflict of interest.

## Publisher's note

All claims expressed in this article are solely those of the authors and do not necessarily represent those of their affiliated organizations, or those of the publisher, the editors and the reviewers. Any product that may be evaluated in this article, or claim that may be made by its manufacturer, is not guaranteed or endorsed by the publisher.

## References

[1] Sung H Ferlay J Segel RL Laversanne M Soerjomataram I. Global cancer statistics 2020: GLOBOCAN estimates of incidence and mortality worldwide for 36 cancers in 185 countries. CA Cancer J Clin. (2021) 71:209–49. 10.3322/caac.21660 33538338

[2] Ferlay J Soerjomataram I Dikshit R Eser S Mathers C Rebelo M. Cancer incidence and mortality worldwide: sources, methods and major patterns in GLOBOCAN 2012. Int J Cancer. (2015) 136:E359–86. 10.1002/ijc.29210 25220842

[3] Arbyn M Castellsague X de Sanjose S Bruni L Saraiya M Bray F. Worldwide burden of cervical cancer in 2008. Ann Oncol. (2011) 22:2675–86. 10.1093/annonc/mdr015 21471563

[4] Ferlay J Rim Shin H Bray F Forman D Mather C Maxwell Parkin D. Estimates of worldwide burden of cancer in 2008: GLOBOCAN 2008. Int J Cancer. (2010) 127:2893–917. 10.1002/ijc.25516 21351269

[5] Safaeian M Solomon D Castle PE. Cervical cancer prevention–cervical screening: science in evolution. Obstet Gynecol Clin North Am. (2007) 34:739–60. 10.1016/j.ogc.2007.09.004 18061867 PMC2762353

[6] Lim JN Ojo AA. Barriers to utilisation of cervical cancer screening in Sub Sahara Africa: a systematic review. Eur J Cancer Care. (2017) 26:12444. 10.1111/ecc.12444 26853214

[7] McFarland DM Gueldner SM Mogobe KD. Integrated review of barriers to cervical cancer screening in sub-Saharan Africa. J Nurs Scholarsh. (2016) 48:490–8. 10.1111/jnu.12232 27434871

[8] Randall TC Ghebre R. Challenges in prevention and care delivery for women with cervical cancer in sub-Saharan Africa. Front Oncol. (2016) 6:160. 10.3389/fonc.2016.00160 27446806 PMC4923066

[9] Chidyaonga-Maseko F Chirwa ML Muula AS. Underutilization of cervical cancer prevention services in low and middle income countries: a review of contributing factors. Pan Afr Med J. (2015) 21:231. 10.11604/pamj.2015.21.231.6350 26523173 PMC4607967

[10] Denny L Quinn M Sankaranarayanan R. Chapter 8: screening for cervical cancer in developing countries. Vaccine. (2006) 24(Suppl.3):S3/71–7. 10.1016/j.vaccine.2006.05.121 16950020

[11] Mukakalisa I Bindler R Allen C Dotson J. Cervical cancer in developing countries: effective screening and preventive strategies with an application in Rwanda. Health Care Women Int. (2014) 35:1065–80. 10.1080/07399332.2014.909433 24750113

[12] Demographic Health Survey. National Statistics Office (NSO). Zomba, Malawi; Calverton, MD: ICF Macro (2012).

[13] Rwanda Biomedical Centre. A First Look at Selected Findings From the 2019-20 RDHS. (2020). Available online at: https://rbc.gov.rw/index.php?id=188 (accessed June 12, 2022).

[14] WHO. Success Factors for Women's and Children's Health. Rwanda. (2015).

[15] Watson-Jones D Baisley K Ponsiano A Lemme F Remes P Ross D. Human papillomavirus vaccination in Tanzanian schoolgirls: cluster-randomized trial comparing 2 vaccine-delivery strategies. J Infect Dis. (2012) 206:678–86. 10.1093/infdis/jis407 22711908 PMC3414230

[16] World Bank. The World Bank in Sierra Leone. Washington, DC: World Bank (2021).

[17] UNDP. Maternal Health. UNDP-SIERRA LEONE: Sierra Leone (2021).

[18] Leone GoS. National Action Plan for Health Security. GoSL: Sierra Leone (2022).

[19] Jemal A Bray F Center MM Ferlay J Ward E Forman D. Global cancer statistics. Cancer J Clinicians. (2011) 61:69–90. 10.3322/caac.20107 21296855

[20] Singh DK Anastos K Hoover DR Burk RD Shi Q Ngendahayo L. Human papillomavirus infection and cervical cytology in HIV-infected and HIV-uninfected Rwandan women. J Infect Dis. (2009) 199:1851–61. 10.1086/599123 19435429 PMC2814215

[21] Makuza JD Nsanzimana S Muhimpundu MA Pace LE Ntaganira J Riedel DJ. Prevalence and risk factors for cervical cancer and pre-cancerous lesions in Rwanda. Pan Afri Medical J. (2015) 22:7116. 10.11604/pamj.2015.22.26.7116 26664527 PMC4662515

[22] WHO. Comprehensive Cervical Cancer Control: A Guide to Essential Practice. Geneva: World Health Organization (2006). 25642554

[23] ICO/IARC. Rwanda Human Papillomavirus and Related Cancers, Fact Sheet 2021. Barcelona: ICO/IARC Information Center on HPV and Cancer (2021).

[24] Ferlay JEM Lam F Colombet M Mery L Piñeros M Znaor A. Global Cancer Observatory: Cancer Today. (2020). Available online at: https://gco.iarc.fr/today (accessed June 8, 2022).

[25] Bruni L Serrano B Mena M Collado JJ Gómez D Muñoz J. ICO/IARC Information Centre on HPV and Cancer (HPV Information Centre). Human Papillomavirus and Related Diseases in Rwanda. Summary Report 22 October 2021. Barcelona: ICO/IARC HPV Information Centre, Institut Català d'Oncologia Spain (2022).

[26] Binagwaho A Ngabo F Wagner CM Mugeni C Gatera M Nutt CS. Integration of comprehensive women's health programmes into health systems: cervical cancer prevention, care and control in Rwanda. Bull World Health Organ. (2013) 91:697–703. 10.2471/BLT.12.116087 24101786 PMC3790215

[27] Ahmed SM Rawal LB Chowdhury SA Murray J Arscott-Mill S. Cross-country analysis of strategies for achieving progress towards global goals for women's and children's health. Bull World Health Organ. (2016) 94:351–61. 10.2471/BLT.15.168450 27147765 PMC4850533

[28] Binagwaho A Wagner CM Gatera M Karema C Nutt CT Ngabo F., Achieving high coverage in Rwanda's national human papillomavirus vaccination programme. Bullet World Health Org. (2012) 90:623–8. 10.2471/BLT.11.097253 22893746 PMC3417784

[29] ICO/IARC. Human Papillomavirus and Related Cancers, Fact Sheet 2021. Barcelona: ICO/IARC (2021).

[30] Bruni L Albero G Serrano B Mena M Collado JJ Gomez D. ICO/IARC infromation Center on HPV and Cancer (HPV Information Centre.). Human Papillomavirus and Related Diseases Report-Sierra Leone. Summary Report 22 October 2021. Barcelona: ICO/IARC (2022).

[31] World Health Organization. Global Report on Trends in Prevalence of Tobacco Use 2000-2025. Geneva (2019).

[32] UN. World Contraceptive Use 2019. Geneva: United Nations (2019).

[33] UNAIDS. Global Data on HIV Epidemiology and Response. Geneva: UNAIDSinfo (2019).

[34] UNAIDS. HIV Sero-Prevalence Study For Key Populations May 2015. Sierra Leone (2015).

[35] McPake B Dayal P Herbst CH. Never again? Challenges in transforming the health workforce landscape in post-Ebola West Africa. Hum Resour Health. (2019).17:19. 10.1186/s12960-019-0351-y 30845978 PMC6407225

[36] Ministry of Health and Sanitation: Sierra Leone. Training and Managing the Health Workers of Tomorrow: New Human Resources Strategy Launhed for Sierra Leone. Ministry of Health and Sanitation: Sierra Leone (2017). p. 2.

[37] Lancet. GBD 2017: a fragile world. Lancet. (2018) 392:1683. 10.1016/S0140-6736(18)32858-7 30415747

[38] Rubagumya F Costas-Chavarri A Manirakiza A Murenzi G Uwinkindi F Ntizimira C. State of cancer control in Rwanda: past, present, and future opportunities. JCO Glob Oncol. (2020) 6:1171–7. 10.1200/GO.20.00281 32701365 PMC7392739

[39] Pratt CP. How breast cancer patients in Sierra Leone are affected by COVID-19. In: Blog, CP Pratt, editor, Sierra Leone: Google (2020). p. 1.

[40] PATH. Global HPV Vaccine Introduction Overview. In Projected and Current National Introductions, Demonstration/Pilot Projects, Gender Neutral Vaccination Programs, and Global HPV Vaccine Introduction Maps. Washington DC (2017).

[41] Zhang J Zhao Y Dai Y Dang L Ma L Yang C. Effectiveness of high-risk human papillomavirus testing for cervical cancer screening in China: a multicenter, open-label, randomized clinical trial. J Am Med Assoc Oncol. (2021) 7:263–70. 10.1001/jamaoncol.2020.6575 33377903 PMC7774051

[42] Preston SH. The changing relation between mortality and level of economic development. Popul Stud. (1975) 29:231–48. 10.1080/00324728.1975.10410201 11630494

[43] Rwanda. Current Health Expenditure Per Capita. World Bank (2018).

[44] WHO. The Global Health Observatory-Health Equity Country Profiles. Geneva (2021).

[45] Economy. The Global Economy-Business and Economic Data for 200 Countries. London (2022).

[46] Carshon-Marsh R Aimone A Ansumana R Swaray IB Assalif A Musa A. Child, maternal, and adult mortality in Sierra Leone: nationally representative mortality survey 2018–20. Lancet Global Health. (2022) 10:e114–23. 10.1016/S2214-109X(21)00459-9 34838202 PMC8672062

[47] WHO, UNFPA, World Bank Group and the United Nations Population Division. Trends in Maternal Mortality: World Health Organization, Maternal Mortality Ratio (Modeled Estimate, per 100,000 Live Births) - Mongolia. (2019).

[48] WHO. Human papillomavirus vaccines: WHO position paper, May 2017. Wkly Epidemiol Rec. (2017) 92:241–68. 28530369

[49] WHO. One-dose Human Papillomavirus (HPV) Vaccine Offers Solid Protection Against Cervical Cancer. (2022). Available online at: https://www.who.int/news/item/11-04-2022-one-dose-human-papillomavirus-(hpv)-vaccine-offers-solid-protection-against-cervical-cancer (accessed June 12, 2022). PMC9280593 35537734

[50] Liu C. Cervical Breast Cancers Screening Project for Women Aged 35–64 Years Are Implementing Smoothly in Ordos. (2016). Available online at: http://www.ordos.gov.cn/gk_128120/shjz/yljz/201603/t20160316_2496165.html (accessed June 12, 2022).

[51] Wang MZ Feng RM Wang S Duan XZ Li D Zhang X. Clinical performance of human papillomavirus testing and visual inspection with acetic acid in primary, combination, and sequential cervical cancer screening in China *Sex Transm Dis*. (2019) 46:540–7. 10.1097/OLQ.0000000000001026 31295223 PMC6911030

[52] Niyonsenga G Gishoma D Sego R Uwayezu MG Nikuze B Fitch M. Knowledge, utilization and barriers of cervical cancer screening among women attending selected district hospitals in Kigali-Rwanda. Can Oncol Nurs J. (2021) 31:266. 10.5737/23688076313266274 34395829 PMC8320790

